# Association and symptom characteristics of irritable bowel syndrome among bronchial asthma patients in Kuwait

**DOI:** 10.4103/1817-1737.58958

**Published:** 2010

**Authors:** Radhakrishna Panicker, Nermina Arifhodzic, Mona Al Ahmad, Seham Ahmed Ali

**Affiliations:** *Al-Rashed Allergy Centre, Ministry of Health, Kuwait*; 1 *Faculty of Science, Kuwait University, Kuwait*

**Keywords:** Asthma, irritable bowel syndrome, allergy

## Abstract

**CONTEXT:**

Excess prevalence of irritable bowel syndrome in asthma has been reported, suggesting a link between these two conditions.

**AIMS:**

To investigate the association between irritable bowel syndrome (IBS) and asthma, and explore the symptoms of IBS among asthma patients in Kuwait.

**SETTINGS AND DESIGN:**

Case control study.

**METHODS:**

In a tertiary center, for allergy and asthma, 138 patients aged 20-65 years, with asthma, diagnosed clinically and by spirometry, were compared with 145 healthy, non-asthmatic controls matched for age, gender and nationality. Cases and controls completed a self-administered questionnaire of irritable bowel syndrome diagnosis (ROME II criteria).

**STATISTICAL ANALYSIS USED:**

The data were analyzed using SPSS software, and proportions were tested with Chi-square or Fisher's test. Odds ratio (OR) with 95% Confidence Interval (CI) were calculated to identify the associated risk factors. The demographic variables were selected for logistic regression analysis.

**RESULTS:**

A significantly large proportion (39.13%) of asthmatics had IBS as compared to 7.93% controls (*P* < 0.001). A higher proportion of females with IBS were observed in cases and controls (74%, 61.54%). IBS was seen in 87% cases using inhalers, and in 13% with additional oral theophylline (*P* < 0.001). As many as 66.6% cases, had IBS with relatively short duration of asthma (1-5 years, *P* < 000). Predominant symptoms of IBS in asthmatics were abdominal discomfort or distension (64.8% vs. 11.5%), (*P* < 0.000, OR = 14.1; 95%CI: 3.748-53.209), bloated feeling of abdomen (74.1% vs. 34.62% (*P* < 0.001, OR = 5.38; 95%CI:1.96-14.84)), increased frequency of stools (63%, *P* < 0.006).

**CONCLUSIONS:**

Irritable bowel syndrome in asthmatics was significantly high, more in the female asthmatics. Abdominal discomfort, persistent bloated feeling, increased frequency of passing stools were the most common IBS symptoms observed.

Irritable bowel syndrome (IBS) is a functional disorder consisting of altered bowel habits, abdominal pain and absence of any detectable organic pathologic process. It is a chronic abdominal symptom complex and is widely recognized as one of the commonest gastrointestinal disorders. Symptoms consistent with IBS are reported by 15-20% of the general population[1] and IBS accounts for 50% of all referrals to gastroenterologists.[2]

Excess prevalence of bronchial hyper-responsiveness has been shown among patients with IBS.[3] A review of the literature reveals that both conditions coexist and they are related. To explore this association, studies have been conducted among asthma patients for IBS, and among IBS patients for asthma, but with conflicting results. There is a shortage of medical literature examining the relationship between IBS and asthma.

Roussos *et al*.[3] evaluated 150 patients with asthma, 130 patients with other pulmonary disorders and 120 healthy subjects. They found that patients with bronchial asthma have an increased prevalence of IBS than the other two groups. An association of gastrointestinal symptoms like IBS and allergic diseases like allergic rhinitis and asthma has been reported in a large case-control study by Nick Powell.[4]

On the other hand, studies conducted by Consuelo Huerta reported only slight increased risk of IBS among asthma patients compared to the general population.[5]

White *et al*. showed an increased prevalence of bronchial hyper-responsiveness in IBS patients and that bronchial hyper-reactivity is the characteristic feature of bronchial asthma.[6] Yazar *et al*., found a higher rate of asthma in IBS patients than in controls and speculated that gastrointestinal system and respiratory system may be affected by a common disorder.[7] In further analyzing for an objective diagnosis of asthma, Ricconi *et al.*, did a methacholine challenge test in IBS patients but found no relationship with asthma.[8] Ju *et al*., concluded a similar finding after conducting a methacholine challenge test in IBS patients and reported that there is no relationship between methacholine challenge test and IBS, but a relationship might exist in a subpopulation of patients whose symptoms worsen by stress.[9]

In view of these conflicting reports about the association of asthma and IBS, we sought to investigate the relationship of IBS in our asthma patients. Although there are reports supporting the high prevalence of IBS in asthma, the individual symptoms of IBS in asthma have not been fully evaluated. To our knowledge, this represents the first study analyzing the predominant gastrointestinal symptoms of IBS among asthma patients in our area.

## Methods

### Patients

A total of 138 asthmatic patients were recruited from the allergy outpatient clinic during the period September 2007 to August 2008. Patients were included in the study if they fulfilled the following inclusion criteria: (1) a physician-diagnosed asthma clinically and (2) evidence of reversibility on spirometry with > than 15% of FEV1 after salbutamol inhalation. Severity was assessed by spirometry, and divided into mild, moderate and severe based on FEV1 measurements (FEV1 60-70% mild, 50-60% moderate, and below 50% severe). All patients were using 1000 mcg of beclamethasone or fluticasone equivalents daily, salbutamol-metered dose inhaler 200-400 mcg as and when needed. Moderate to severe asthmatics were on long-acting beta agonist salmeterol 50 mcg twice daily along with inhaled steroids. Some patients were on oral theophylline (sustained action theophylline 250 mg twice daily) in addition to inhaled medications to control their asthma. No patients were on oral steroids.

Exclusion criteria included: (1) patients with acute asthma exacerbation or respiratory infection. (2) Any history of abdominal, organic bowel disease, or abdominal surgery. (3) Any history of weight loss. (4) History of diabetes, hypertension, or cardiac diseases. (5) Any other medication except for bronchial asthma. Gender and age-matched healthy controls (n = 145) without any history of any respiratory, cardiac, abdominal or surgical history were included in the study. All asthma and controls answered an IBS questionnaire (ROME II criteria). Both cases and controls signed an informed consent to participate in the study. A research ethics approval was obtained for this study from the higher research ethics approval committee.

### IBS evaluation

All patients and healthy controls were selected as having IBS after answering the IBS questionnaire (ROME II criteria) The individual symptoms of IBS were then analyzed and evaluated for their significance. Physical examination of abdomen was normal in all the selected patients and controls.

### Rome II criteria

Rome II criteria[10] are considered as the gold standard in diagnosing IBS. This requires the existence of abdominal discomfort or pain for at least 12 weeks, which need not be consecutive, in the preceding 12 months. The discomfort or pain should not be explained on the basis of structural or biochemical abnormalities and that has at least two of the following three features: (1) pain is relieved with defecation. (2) the onset is associated with a change in the frequency of bowel movements, and (3) its onset is associated with changes in stool consistency, discomfort or pain. The Rome diagnostic criteria for functional abdominal pain and abdominal discomfort are widely used in research as well as practice. Population-based surveys using Rome II criteria have shown substantial agreement between Rome I and Rome II criteria for IBS[11]

The questionnaire was translated from English into Arabic by a native Arabic physician and was reviewed by another native Arabic physician and an Arabic non-physician, before being administered to patients and controls. Diagnosis of IBS was based on the Rome II criteria.

### Statistical analysis

The data, for cases and controls, was analyzed for demographic and clinical variables, and compared to find any association or significant differences in proportions using Chi-square or Fisher's test. Odds ratio (OR) with 95% Confidence Interval (CI) were calculated to identify the associated risk factors. A probability level, *P* < 0.05 was used as the criterion for statistical significance. Logistic regression analysis was applied to the demographic variables to assess its association of IBS in asthma patients. Statistical package for social sciences (SPSS, version 17, 2005; SPSS Inc. Chicago, IL, USA) was used for data management and analysis.

## Results

**Figure 1:**
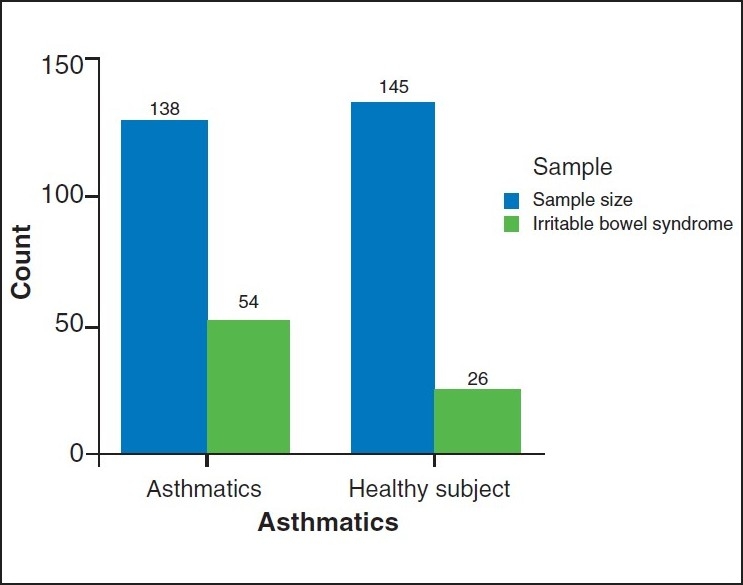
The prevalence of IBS in asthmatics and control subjects

IBS was found in 54 out of 138 asthma patients (39.13%) and 26 out of 145 controls (17.93% *P* < 0.001) [Table 1, Figure 1].

**Table 1:** The prevalence of IBS in asthmatics and control subjects

	Asthmatics	Healthy subjects	*P* value
Sample size	138	145	0.000
Total IBS	54 (39.13%)	26 (17.93%)	

IBS: Irritable bowel syndrome

The demographic data of asthmatic patients and matched healthy controls are shown in Table 2. Logistic regression analysis did not show any significant association between demographic variables as risk factors for IBS in asthma. However, the frequency of IBS in females was higher than in males, both in cases and controls.

**Table 2:** Demographic variables of IBS in asthma and control

Factors	Asthma (frequency)		Control (frequency)		*P* value OR (95%CI)
	N=54	(%)	N=26	(%)	
Age					
20-30	14	25.93	8	30.77	0.338
					2.693
					(0.355-20.443)
31-40	21	38.88	8	30.77	0.168
					4.107
					(0.552-30.543)
41-50	17	25.92	7	26.92	0.177
					4.169
					(0.524-33.190)
51-60	5	9.26	3	11.54	
Gender					
M	14	25.93	10	38.46	0.320
					0.592
					(0.211-1.665)
F	40	74.07	16	61.54	
Nationality					
Kuwaiti	40	74.07	18	69.23	0.550
					1.400
					(0.465-4.214)
Not-Kuwaiti	14	25.93	8	30.77	
Smoker					
Smoker	12	22.22	6	23.08	0.998
					0.998
					(0.316-3.153)
Non-smoker	42	77.77	20	76.92	
Treatment					
ISt+bd*	47	87.03			0.346
IS+bd+th†	7	12.96			
Asthma					
Duration- years					
1-5	36	66.66			0.000
5-10	11	20.3			
More than10	7	12.96			
Asthma severity					
Mild	32	59.26			0.883
Moderate	15	27.78			
Severe	7	12.96			

* ISt + bd: Inhaled steroid and bronchodilators

† ISt+bd+th: Inhaled steroid and bronchodilator and theophylline

Although statistically not significant, the trend shows that IBS is more frequent when the women are suffering from asthma (74.7% vs. 57.4% in controls). IBS was found more among Kuwaitis in both cases and controls as compared to non-Kuwaitis. We found that non-smokers were more affected by IBS (78% of cases and 77% of controls).

Majority of patients (87%) were using inhaled steroids and inhaled salbutamol and only seven patients (13%) used oral theophylline along with inhaled medications. There was no significant difference in the presence of IBS between those on oral theophylline and others. With respect to duration of asthma, IBS was more prevalent (67%) among those with short asthma duration (1-5 years) compared to longer duration of asthma 5-10 years (20%). IBS was found to be the least in those with more than 10 years of asthma duration (12%). These findings are consistent with the observation, that IBS in the general population showed a decline in trend, as the age advances [Table 2].

Table 3 shows the IBS symptoms analysis. Abdominal pain or discomfort was found in 65% among asthmatics and in only 12% of controls (*P* < 0.05). Bloated feeling of abdomen was found in 74% of cases with IBS and 34% in controls (*P* < 0.005). Passing stools more than three times per day was more common in cases as compared to controls *P* < 0.006).

**Table 3:** Irritable bowel syndrome symptom analysis

Symptoms		Asthma		Control		*P* value OR (95%CI)
			
		Frequency	%	Frequency	%	
Abdominal pain/discomfort	Yes	35	64.81	3	11.5	0.000*
						(14.123)
						(3.748,53.209)
	No	19	35.19	23	88.46	
Abdominal pain relieved by passing stools	Yes	25	46.29	9	34.62	0.322
						1.628
						(0.618,4.291)
	No	29	53.70	17	65.38	
Stool more than 3/day	Yes	20	62.96	24	92.31	0.006
						7.059
						(1.506,33.079)
	No	34	37.03	2	7.69	
Stool less than 3/week	Yes	17	31.48	6	23.08	0.437
						1.532
						(.521,4.501)
	No	37	68.52	20	76.92	
Loose/hard or watery stools	Yes	25	46.29	12	46.15	0.990
						1.006
						(0.393,2.571)
	No	29	53.70	14	53.85	
Mucus in stools	Yes	9	16.67	6	23.08	0.491
						0.667
						(0.209,2.126)
	No	45	83.33	20	76.92	
Bloated feeling of abdomen	Yes	40	74.07	9	34.62	0.001*
						5.397
						(1.963,14.840)
	No	14	25.93	17	65.38	

Other variables for the diagnosis of IBS in the Rome II Criteria like passing stools less than three per week, hard stools or stools with mucous were not statistically significant from those among healthy controls.

## Discussion

Distension or discomfort are the commonly encountered abdominal symptoms among asthma patients and are either ignored or considered as drug-induced gastritis from oral medicines like theophylline or as a result of gastro-esophageal reflux disease (GERD).

In a study by Kennedy *et al*., they found that IBS, GERD, and symptomatic bronchial hyper-reactivity occur more frequently together than expected, and these conditions are independently associated with each other.[12]

Babak Amra *et al.*, reported similar observations that symptoms of IBS and asthma occur more frequently together and are independently associated with each other.[13]

In this study we found a significantly high percentage of IBS (*P* < 0.005) among asthmatics as compared to matched healthy subjects, supporting the association of IBS in asthma patients.

In our study, IBS was more common in asthmatic women, both in cases and controls. The high prevalence of IBS among women is a well documented, unexplained feature of all functional gastrointestinal disorders. Emotional disturbance may contribute to the high prevalence of IBS in female asthmatics. There are studies showing that asthmatic females are more psychologically distressed than asthmatic males.[14] Stress, anxiety and psychosomatic factors are all implicated in asthma and in IBS, and these may influence the pathogenesis of IBS in asthma. Our finding of passing frequent stools (more than three per day) in asthmatics having IBS may be explained on the basis of this anxiety and stress.

The frequency of IBS in asthma was less as the duration of asthma was long, and this finding of declining trend was quite interesting. IBS in the general population also decreases as the age advances. We found that the existence of IBS among asthmatics of young age (31-60 years) was high as compared to older age (> 60 years). Ekici compared two groups of asthmatic patients with ages >60 years and < 60 years with age-matched controls. The prevalence of IBS was high among asthmatics with age <60 years.[15]

Our finding is consistent with this observation and supports that most of our asthma patients with IBS (68.4%) were in the age range of 31-50 years.

As the symptoms of IBS are nonspecific, the diagnosis is usually made by exclusion of other gastrointestinal conditions. Previous studies conducted in asthma patients were mainly focused on the prevalence of IBS using the definition of Rome II criteria. However, the predominant abdominal symptoms of IBS in asthmatics were not evaluated to our knowledge.

IBS is a symptom complex with diffuse abdominal symptoms that vary from person to person. In this study we analyzed all the abdominal symptoms as per the Rome II criteria. We found that abdominal discomfort with or without abdominal pain was the most common and significant symptom among asthmatics (*P* < 0.001). Bloated feeling in the abdomen was a predominant symptom among all asthma patients having IBS (*P* < 0.005). This might be explained on the basis of the gaseous distension of intestines, possibly due to the abnormal gastrointestinal motility. Abdominal distension and bloated feeling are characteristic features of IBS. It has been shown that there is an altered contractility and smooth muscle tone in patients with IBS and asthma.[16] A large proportion of patients with IBS have impaired transit and tolerance of intestinal gas loads. This anomaly may represent a possible mechanism of IBS symptoms, specifically pain and bloating. The gastrointestinal symptoms might indicate a common pathology of smooth muscle dysfunction in asthma and IBS. Jones *et al*., postulated a generalized disorder of bronchial, gastro-intestinal and other smooth muscles.[17] On the other hand, in asthma patients with GERD, there is an evidence of autonomic dysfunction with primarily hyper-vagal responsiveness.[18]

There was no difference in the prevalence of IBS in patients with and without oral theophylline (52% of cases vs. 48% of controls). Cole *et al*. studied the association between oral steroids and IBS in a cohort of people with asthma and without asthma. They found no association between oral steroids and IBS among people with asthma.[19] Our study is consistent with this finding and oral medication may not be the cause of abdominal symptoms. The inhaled bronchodilators are bronchial smooth muscle relaxants and their effects on intestinal smooth muscles are unlikely, as not all patients on inhaled bronchodilators have IBS. The role of inhaled medications in the pathogenesis of IBS is not yet known.

Smart *et al*.,[20] proposed that IBS might be secondary to autonomic imbalance possibly due to hypothalamic dysfunction. Even though it is not proved, autonomic imbalance might produce broncho-spasm as a response to inhaled stimulus.[21]

The autonomic nervous system plays an important role in the pathogenesis of asthma. The autonomic nervous system regulates the mucous secretion, tonicity of bronchial smooth muscles, blood flow, micro-vascular permeability, and functions of inflammatory cells.[22] Indirect support for the evidence of autonomic imbalance in IBS has been well established.[23] Acetylcholine secreted from the vagus lead to contraction of bronchial smooth muscle by stimulation of muscarinic receptors 3 (M3). Extra - intestinal causes, like urinary symptoms and sexual dysfunction, have been found in IBS showing a generalized smooth muscle contractility or altered neural regulation or autonomic imbalance.[24]

On the basis of animal studies,[25] a relationship between inflammation and altered gut motility has been proposed. Mast cells have been found in increased numbers in the gut in some IBS patients. The dysregulation of mast cells results in changes in muscle contraction and in enteric nerve excitability.[26,27] Degranulation of mast cells leading to the release of inflammatory mediators is known to cause hyper-reactive airways in allergic asthma. Therefore the possibility of a common altered immune system should also be considered in the pathogenesis of IBS in asthma. To postulate a common etiological agent like food allergy to cause asthma and IBS, Duygu Ozol *et al*. studied the role of food allergy, and reported that there was no significant association between asthma-related parameters, IBS and food allergy.[28]

## Conclusion

This study showed that IBS among our asthma patients was significantly high as compared to non-asthmatics. Abdominal discomfort and bloated feeling of abdomen or increased frequency of loose stools in asthma patients should raise a suspicion of IBS. This in turn might be addressed while treating asthma, for better health care. The link in the pathogenesis between asthma and IBS needs further studies to document the role of smooth muscle dysfunction.
